# Synaptic GAP and GEF Complexes Cluster Proteins Essential for GTP Signaling

**DOI:** 10.1038/s41598-017-05588-3

**Published:** 2017-07-13

**Authors:** Brent Wilkinson, Jing Li, Marcelo P. Coba

**Affiliations:** 1 Zilkha Neurogenetic Institute, Keck School of Medicine, University of Southern California, Los Angeles, CA 90033 USA; 2 Department of Psychiatry and Behavioral Sciences, Keck School of Medicine, University of Southern California, Los Angeles, CA 90033 USA

## Abstract

GTPase-activating proteins (GAPs) and guanine exchange factors (GEFs) play essential roles in regulating the activity of small GTPases. Several GAPs and GEFs have been shown to be present at the postsynaptic density (PSD) within excitatory glutamatergic neurons and regulate the activity of glutamate receptors. However, it is not known how synaptic GAP and GEF proteins are organized within the PSD signaling machinery, if they have overlapping interaction networks, or if they associate with proteins implicated in contributing to psychiatric disease. Here, we determine the interactomes of three interacting GAP/GEF proteins at the PSD, including the RasGAP Syngap1, the ArfGAP Agap2, and the RhoGEF Kalirin, which includes a total of 280 interactions. We describe the functional properties of each interactome and show that these GAP/GEF proteins are highly associated with and cluster other proteins directly involved in GTPase signaling mechanisms. We also utilize Agap2 as an example of GAP/GEFs localized within multiple neuronal compartments and determine an additional 110 interactions involving Agap2 outside of the PSD. Functional analysis of PSD and non-PSD interactomes illustrates both common and unique functions of Agap2 determined by its subcellular location. Furthermore, we also show that these GAPs/GEFs associate with several proteins involved in psychiatric disease.

## Introduction

Small GTPases are molecular switches that can rapidly interconvert between two conformational states, depending on association to GTP or GDP^1^. The cellular actions of GTPases are frequently initiated by GTP binding, enabled by guanine nucleotide exchange factors (GEFs), and finalized by GTP hydrolysis facilitated by GTPase-activating proteins (GAPs)^1, 2^. The large number of existing GTPases require a multitude of GEFs and GAPs that utilize a variety of combinatorial mechanisms to achieve functional specificity^3, 4^. These proteins not only need to respond to different inputs, but also direct their output to specific small GTPases. Proteins containing GAP and GEF domains are usually distributed in families containing a variety of protein domains^4, 5^. These domains are arranged in multi-domain architectures including a family specific GAP/GEF domain together with domains that have the capacity to respond to different chemical messages and associate in protein complexes through protein-protein interactions^4, 5^.

Small GTPases can regulate a variety of cellular functions. In neurons, they have been shown to be involved in different aspects of synaptic function such as regulation of the actin cytoskeleton, spine remodeling, and synaptic plasticity^6–9^. Proteomics analyses have localized a number of GAPs and GEFs at the postsynaptic specialization of glutamatergic excitatory neurons known as the postsynaptic density (PSD)^10, 11^. The PSD contains a collection of more than 1500 proteins arranged in macromolecular complexes and many of these proteins have been linked to a variety of brain disorders^11–16^. PSD protein complexes link glutamate receptors to downstream signaling pathways through a number of scaffold proteins with multi-modular protein domain architectures^11, 13^. However, it is not known how synaptic GAPs and GEFs are organized within this signaling machinery, if they have overlapping binding partners, and if they also associate to brain disease risk factors.

Here, using immmunoisolation and high performance liquid chromatography tandem mass spectrometry (HPLC-MS/MS), we determined the interactomes of the RasGAP Syngap1, the RhoGEF Kalirin, and the ArfGAP Agap2 in PSD fractions of adult mouse cortex. These proteins are able to interact with each other at the PSD and have also been described to be involved in intellectual disability and psychiatric disease^17–21^. We determined 281 protein interactions within the PSD involving these proteins. We described the functional properties and organization of these complexes, along with their role in the organization of GAP and GEF families within the PSD. Because synaptic proteins may function within multiple subcellular locations, we utilized Agap2 as an example of this scenario and determined the interactome of Agap2 in non-PSD fractions, which involved an additional 110 interactions. By comparing domain architecture and binding interactions in different subcellular fractions, we illustrate both common and unique functions of Agap2 based on its cellular localization. With Agap2, Kalirin, and Syngap1 being implicated in contributing to the risk of psychiatric disease, we also show that they interact with and cluster proteins involved in autism spectrum disorder (ASD), schizophrenia (SCZ), and intellectual disability (ID).

## Results

### Distribution of GAPs and GEFs within the postsynaptic density

**Figure 1:**
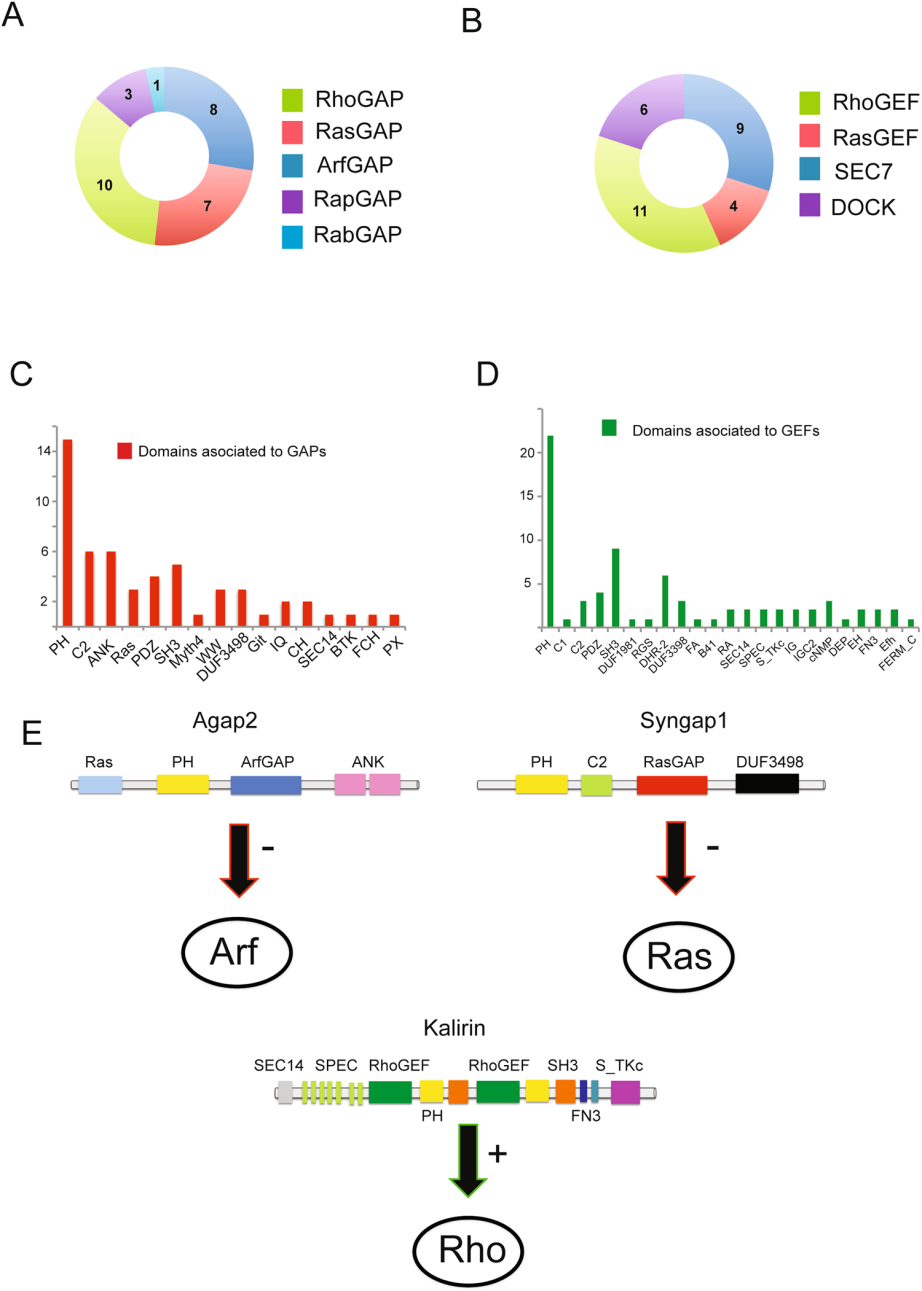
Distribution of GAPs and GEFs at the PSD. (**A**) Distribution of GTPase activating proteins (GAPs) at the postsynaptic density (PSD). (**B**) Distribution of guanine nucleotide exchange factors (GEFs) at the PSD. (**C**) Distribution of protein domain architecture of GAPs at the PSD. (**D**) Distribution of protein domain architecture of GEFs at the PSD. (**E**) Domain architecture of the GAP and GEF proteins used in this study. Abbreviations: ANK, Ankryin repeats; B41, Band 4.1 homologues; BTK, Bruton’s tyrosine kinase cys-rich motif; C1, protein kinase C conserved region 1 domain; C2, protein kinase C conserved region 2 domain; CH, Calponin homology domain; cNMP, Cyclic nucleotide-Monophosphate binding domain; DEP, domain found in disheveled, Egl-10, and Pleckstrin; DHR-2, Dock Homology Region 2; DOCK, Dedicator of cytokinesis; DUF, domain of unknown function; Efh, EF-hand, calcium binding motif; EH – Eps15 Homology domain; FA, FERM Adjacent; FCH, Fes/CIP4 Homology domain; FERM_C, FERM C-terminal PH-like domain; FN3, Fibronectin type 3 domain; GAP, GTPase activator protein; GEF, Guanine nucleotide exchange factor; Git, Helical motif in the GIT family; IG, Immunoglobulin; IGC2 – Immunoglobulin C-2 Type; IQ, Calmodulin-binding motif containing conserved isoleucine (I) and glycine (Q) residues; Myth4. Domain in Myosin and Kinesin tails; PDZ, domain present in PSD-95, Dlg, and ZO-1/2; PH, Pleckstrin homology domain; PX, PhoX homologous domain; Ras, Ras subfamily of small GTPases; RGS, regulator of G-protein signaling; SEC14, domain in homologues of S. cerevisiae Sec14p; SEC7, domain in homologues of S. cerevisiae Sec7; SH3, Src Homology 3 domain; SPEC, Spectrin repeats; S_TKc, serine/threonine protein kinases catalytic domain; WW, domain with 2 conserved tryptophan (W) residues.

In order to explore the PSD signaling landscape of postsynaptic GEF and GAP proteins, we first determined the distribution of GAP and GEF protein domains within PSD proteins. We extracted protein domains from SMART and Pfam databases^22, 23^ and mapped them to 1524 PSD proteins isolated from mouse cortex and identified by HPLC-MS/MS (Supplementary Table S1). From this, we determined 59 PSD proteins containing GAP and GEF domains including 29 GAPs and 30 GEFs (Supplementary Table S2). GAP proteins were distributed among the subfamilies of GTPases, including 10 RhoGAPs, 8 ArfGAPs, 7 RasGAPs, 3 RapGAPs and 1 RabGAP (Fig. 1A). Although RabGAP domain containing proteins are the most abundant within the family of mammalian GTPases^4^, the fact that the Arf, Ras, and Rho GAPs comprise 86% of the total GAPs within the PSD suggests that PSD signaling mechanisms may preferentially use these three sub-families (Fig. 1A). GEF domain containing proteins had a more even distribution with RhoGEF domains (37%) being the most abundant GEF family members at the PSD, followed by SEC7 (30%), DOCK (20%) and RasGEF (13%) (Fig. 1B).

One characteristic of the mammalian PSD, is the organization of signaling proteins in macromolecular protein complexes^11–13^. These protein complexes use scaffold proteins as master-organizers of protein interactions, based on their multi-modular protein domain composition^13, 24^. Several proteins containing GAP and GEF domains also contain a variety of other protein domains, forming multi-domain architectures^5^. Therefore, we determined the protein domain composition of GAP and GEF proteins in the PSD (Supplementary Table S2). While PH domains were the most abundant domain co-occurring with GAPs and GEFs, we also determined the presence of a variety of protein domains whose main function is to organize protein-protein interaction modules (Fig. 1C and D). Therefore, the co-occurrence of GAPs and GEFs with protein domains such as SH3, WW, ANK and PDZ suggests that PSD GTPases and exchange factors can also be associated with and incorporated into PSD protein complexes.

### Interactomes of Agap2, Syngap1, and Kalirin at the postsynaptic density

**Figure 2:**
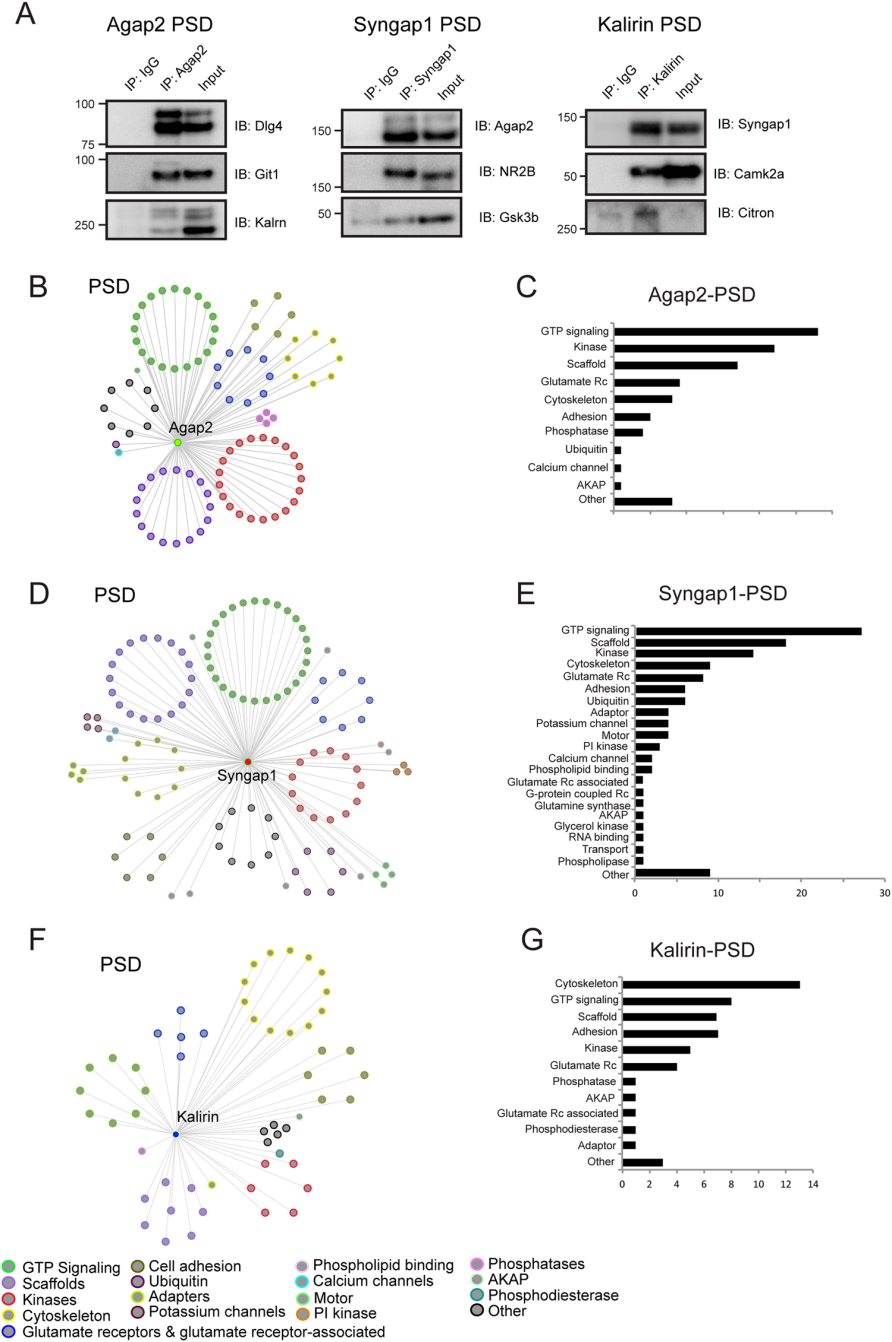
Interactomes of the PSD GAP/GEF proteins, Agap2, Syngap1, and Kalirin. (**A**) Cropped images of immunoprecipitation followed by western blot for interactors of Agap2 (left), Syngap1 (middle), and Kalirin (right) that were identified via HPLC-MS/MS. (**B**–**G**) Interactome of each respective protein is clustered by protein function which is quantified to right. (**B**,**C**) Agap2, (**D**,**E**) Syngap1, (**F**,**G**) Kalirin.

We have previously reported that the RasGAP family member and intellectual disability gene, Syngap1, associates with both Kalirin (RhoGEF) and Agap2 (ArfGAP) in the CA1 area of mouse hippocampus^11^. These three proteins have also been linked to a variety of psychiatric conditions^17–21^ suggesting that they might share a set of common protein interactions within the PSD. Therefore, we immunoprecipitated and isolated the PSD interactomes of Agap2, Syngap1, and Kalirin from adult mouse cortex (Fig. 1E) and determined protein interactions by HPLC-MS/MS as previously described^11^. Here, we identified 104, 124, and 53 interactions in Agap2, Syngap1 and Kalirin PSD protein complexes, respectively (Fig. 2, Supplementary Table S3). Protein interactions for each interactome where then validated by immunoisolation and western blotting assays (Fig. 2A, Supplementary Fig. S1).

We first focused on analyzing different functional groups associated to each interactome individually. Clustering the interactors of the PSD GAPs and GEFs by function shows that Agap2 and Syngap1 were mainly associated to PSD scaffolds, protein kinases, and proteins involved in GTP signaling (Fig. 2B–E, Supplementary Figs S2 and S3, Supplementary Table S3). Although the interactors of Kalirin were also proteins involved in GTP signaling, kinases, and scaffolds, they also included a large number of cytoskeletal proteins in accordance with the role of Kalirin in regulating dendritic spine morphology through RhoGEF activity (Fig. 2F and G, Supplementary Fig. S4)^25, 26^. Thus, Agap2, SynGAP1 and Kalirin share three main functional associations through: (a) interactions to the core-scaffold machinery of the PSD, (b) association with PSD kinases, and (c) clustering of proteins involved in GTP signaling mechanisms.

**Figure 3:**
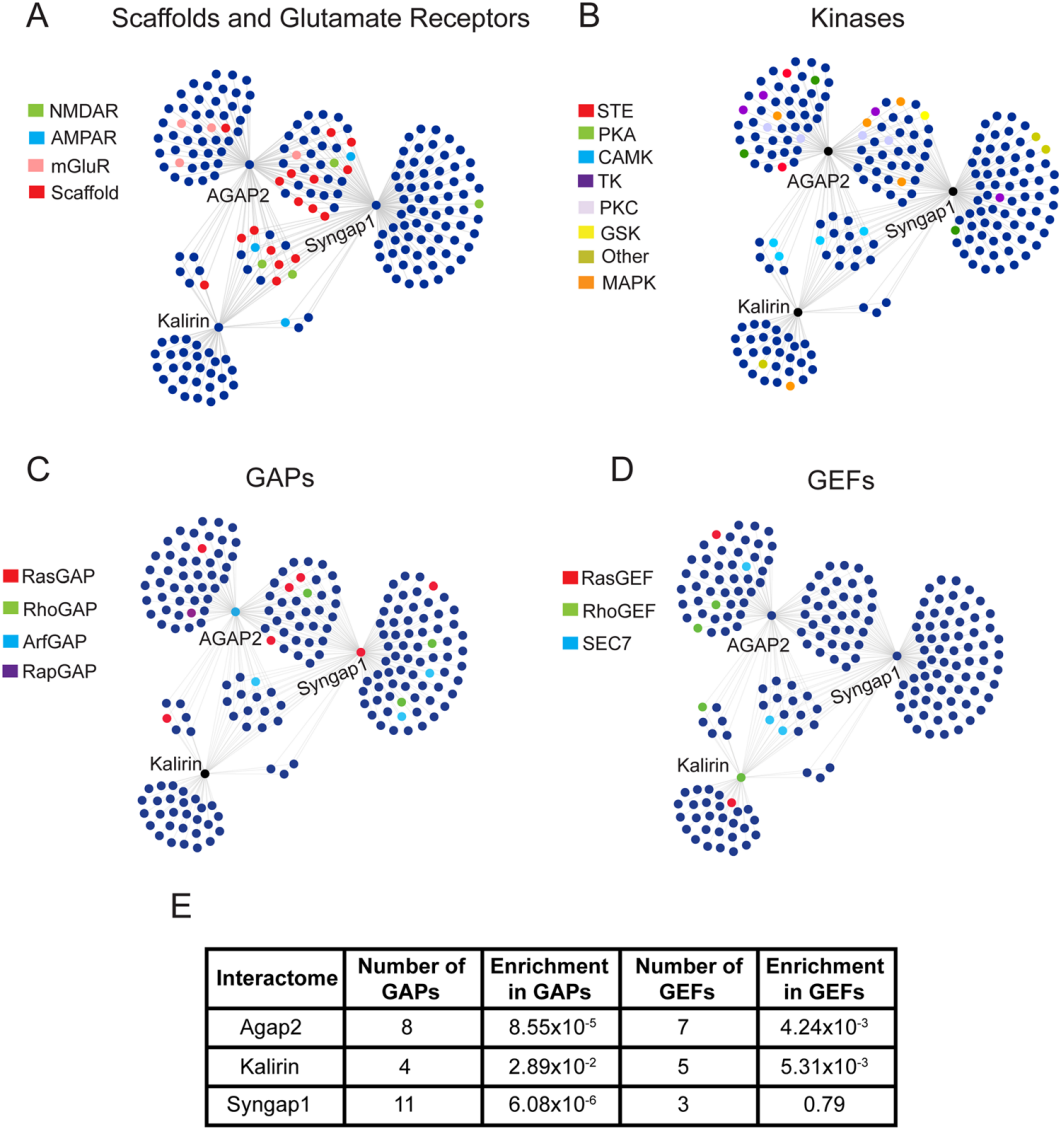
Distribution of Signaling Molecules within PSD Networks. Figure shows the distribution of several different classes of signaling molecules within the three PSD interactomes including: (**A**) Scaffolding proteins and glutamate receptors, (**B**) kinases, (**C**) GAPs, and (**E**) GEFs. (**F**) Enrichment of GAPs and GEFs within the PSD interactomes. P-values represent the results of one-tailed fisher exact tests followed by the Bonferroni correction for multiple comparisons. Abbreviations: CAMK, Ca^2+^/Calmodulin-dependent protein kinase; GAP, GTPase activator protein; GEF, guanine nucleotide exchange factor; GSK, glycogen synthase kinase; MAPK, mitogen-activated protein kinase; SEC7, domain in homologues of S. cerevisiae Sec7; STE, homologs of Ste7, Ste11, and Ste20; TK, tyrosine kinase; PKA, protein kinase A; PKC, protein kinase C.

Similarly to Syngap1, Agap2 and Kalirin also associate with the core-scaffold machinery of the PSD, composed of MAGUKs (Dlg1-4), DLGAPs (Dlgap1-4) and SHANKs (Shank1-3) (Figs 2 and 3A, Supplementary Table S3, Supplementary Fig. S5). This association thus links PSD GAPs and GEFs to NMDA and AMPA ionotropic glutamate receptors through the PSD core-scaffolds, while also binding to metabotropic glutamate receptors (Supplementary Table S3).

While all three PSD protein complexes were found to associate with kinases belonging to an array of protein families, the three complexes shared protein interactions with members of the CAMK family of protein kinases such as the Camk2a and Camk2b kinases (Fig. 3B, Supplementary Fig. S5, Supplementary Table S3). This in accordance with the modulation of Syngap1, Kalirin and Agap2 phosphorylation by Camk2a^11, 26–28^. We recently reported that the induction of LTP increases Agap2 and Syngap1 phosphorylation at multiple CamkIIa phosphosites^11^. Moreover, induction of LTP also induced the increase of Akt1 phsophorylation sites in Agap2^11^, which is reflected in the diverse set of kinases associated to Agap2 (Fig. 3B, Supplementary Fig. S5, Supplementary Table S3). Some kinases, including MAPK and PKC kinases, were shared solely between Syngap1 and Agap2 in accordance with the role of Syngap1 role in the modulation of MAPK families^9, 27^. However, Kalirin was found to associate with a decreased number of kinases, suggesting a more restricted role, not as a modulator of kinases but as a substrate.

In addition to the scaffolds and kinases associated with the PSD GAP/GEF proteins, Syngap1, Agap2 and Kalirin complexes are highly associated with proteins involved in signaling cascades such as GTP signaling (Fig. 2). These three complexes are associated with a multitude of GAP/GEF proteins (Fig. 3C and D, Supplementary Fig. S5) and we hypothesized that they might associate with more GAPs/GEFs than would be expected by chance. Within the PSD there are a total of 29 GAPs and 30 GEFs (Supplementary Table S2). Within the Agap2 interactions we found a total of 9 GAPs and 7 GEFs which are highly enriched relative to the PSD (P = 8.55 × 10^−5^ and P = 4.24 × 10^−3^, respectively, Bonferroni corrected). The same was true for Kalirin complexes (P = 0.029 and P = 5.31 × 10^−3^ for GAPs and GEFs, respectively). While Syngap1 complexes were highly enriched in GAP proteins (P = 6.08 × 10^−6^), they lacked enrichment within GEF proteins (P = 0.79) (Fig. 3E). Overall, this shows that GAPs and GEFs are preferentially organized in multi-GAP and GEF family complexes and not isolated in individual protein interactions at the PSD.

In addition to analyzing Agap2, Kalirin, and Syngap1 complexes by protein function, we also performed over-representation analysis to test for enrichment of protein domains using the SMART database annotation^22^ (Supplementary Table S4). The strong association with the PSD core-scaffolding machinery is reflected through significant enrightment in SH3 domains, characteristic of PSD protein-protein interactions^10^, while the association with NMDA and AMPA glutamate receptors is reflected through enrichment of PBPe (glutamate Rc) domains (P < 0.05 for SH3 and PBPe domain enrichment within all complexes, Bonferroni corrected). Within the Kalirin complex, the SPEC (spectrin) domain has the highest enrichment, corrosponding to the increased number of cytoskeletal interactors (P = 5.02 × 10^−4^). The enrichment of S_TKc (serine/threonine kinase) and RasGAP domains in Agap2 PSD complexes (P = 1.63 × 10^−6^ and 3.75 × 10^−4^ for S_TKc and RasGAP, respectively) and RasGAP domains within Syngap1 (P = 0.03) confirms the clustering of multiple kinases families and RasGAP proteins in these complexes. Moreover, Agap2 is also eniched in the G_alpha domain (P = 1.64 × 10^−5^), corresponding to the association of Agap2 to 6 out of the 10 G-protein alpha subunits localized within the PSD (Supplementary Table S4).

### Differential interactions of Agap2 between PSD and non-PSD compartments

**Figure 4:**
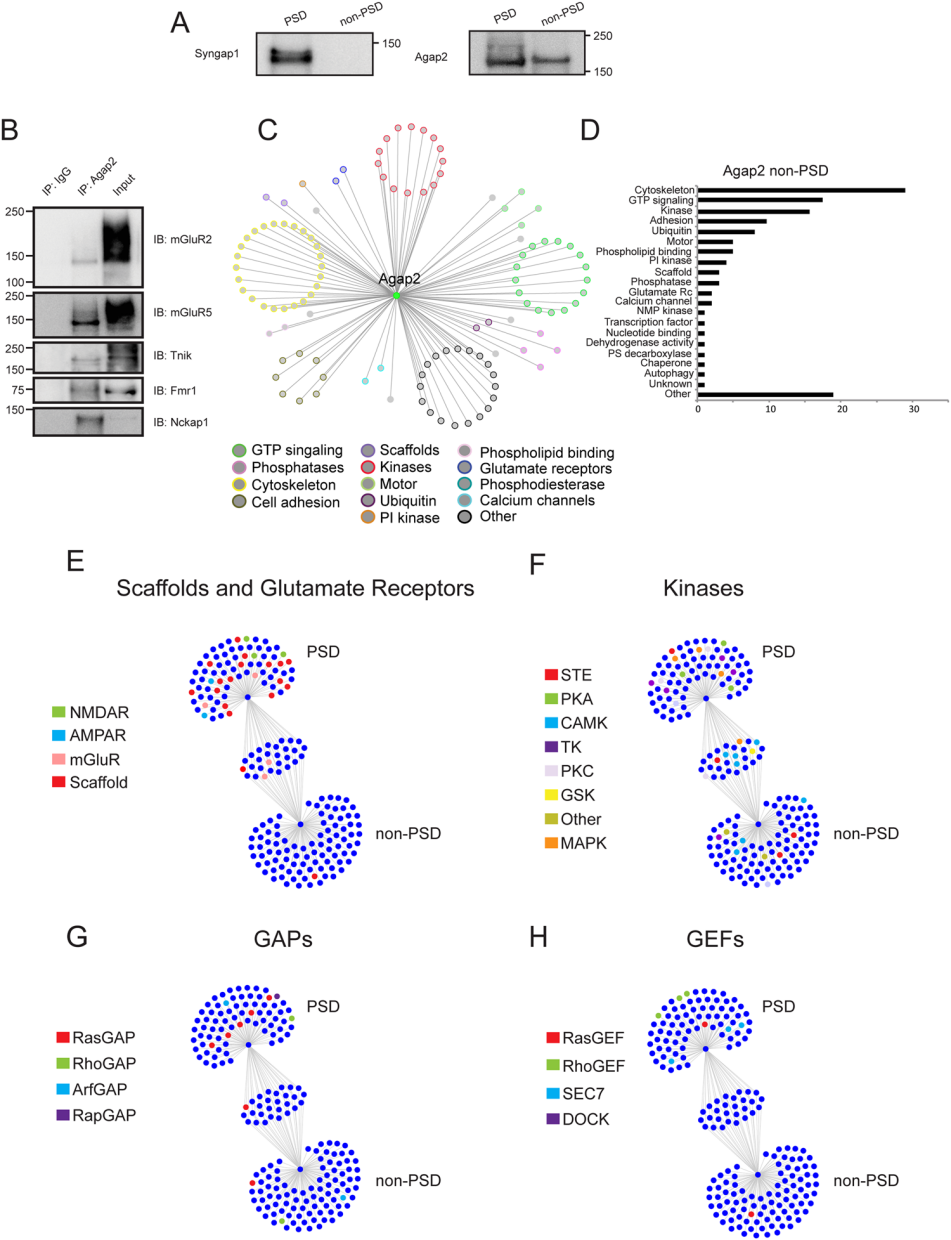
Differential Interactions and Functions of PSD vs Non-PSD Agap2. (**A**) Cropped images of immunoprecipation followed by western blot of Syngap1 and Agap2 post PSD enrichment in adult mouse cortex. Syngap1 is restricted to the PSD fraction while Agap2 is present in both PSD and non-PSD fractions. (**B**) Cropped images of immunoprecipitation followed by western blot for interactors of Agap2 in non-PSD fractions that were identified via HPLC-MS/MS. (**C**) Non-PSD Agap2 interactome clustered by protein function. (**D**) Quantitation of Agap2 interactome by protein function. (**E**–**H**) Comparison of Agap2 PSD interactome versus Agap2 non-PSD interactome illustrating the distributions of (**E**) scaffolding proteins and glutamate receptors, (**F**) Kinases, (**G**) GAPs, and (**H**) GEFs. Abbreviations: CAMK, Ca^2+^/Calmodulin-dependent protein kinase; GAP, GTPase activator protein; GEF, guanine nucleotide exchange factor; GSK, glycogen synthase kinase; MAPK, mitogen-activated protein kinase; SEC7, domain in homologues of S. cerevisiae Sec7; STE, homologs of Ste7, Ste11, and Ste20; TK, tyrosine kinase; PKA, protein kinase A; PKC, protein kinase C.

GAPs and GEFs can also localize in neuronal compartments apart from the PSD^29–32^. Contrary to Syngap1, Agap2 can also localize in non-PSD compartments. Biochemical fractionation shows the presence of Agap2 within both triton soluble fractions^30^ and PSD preparations (Fig. 4A, Supplementary Fig. S6). Thus, we selected Agap2 as an example to compare protein interactions from GAP/GEF complexes between PSD and non-PSD fractions. Therefore, we isolated Agap2 complexes from triton-soluble fractions in addition to PSD preparations^33^ and determined protein interactions by HPLC-MS/MS. Here, we were able to confidently identify 110 Agap2 interacting proteins in the non-PSD fractions (Supplementary Table S3). We also validated a number of novel interactions via immunoprecipitation followed by western blot (Fig. 4B, Supplementary Fig. S6).

Similar to the Agap2 PSD interactome, Agap2 in non-PSD fractions interacted with a large number of kinases and proteins involved in GTP signaling (Fig. 4C and D, Supplementary Fig. S7). However, only 30 interactions were shared with Agap2 interacting proteins within the PSD fraction, corresponding to 75% unique interactions in the non-PSD fraction. As with the PSD interactomes, we chose to compare the Agap2 PSD versus non-PSD interactomes with respect to scaffolding proteins and glutamate receptors, kinases, followed by the associated GAPs and GEFs (Fig. 4E–H, Supplementary Fig. S8).

Functional analysis of interacting partners within Agap2 non-PSD complexes shows that the high association with kinases and GTP signaling proteins remains, but scaffolding protein associations decrease in accordance with a lack of the PSD core scaffolding machinery in non-PSD compartments (Fig. 4E, Supplementary Fig. S8). Although NMDA and AMPA glutamate receptors can also localize at non-PSD compartments, Agap2 in unable to connect to ionotropic glutamate receptors here. While some scaffolding molecules that are not solely localized to the PSD remain, there is an absence of the core-scaffolding molecules of the PSD. This suggests that the link to PSD core-scaffolds is essential to connect Agap2 to AMPA and NMDAR receptors. However, there is a common association of several metabotropic glutamate receptors with Agap2 in both fractions as metabotropic glutamate receptors can localize to peri-PSD sites and they can directly bind to Agap2 in binary protein interactions^34^ (Fig. 4E, Supplementary Fig. S8).

In contrast to Agap2 within the PSD, Agap2 residing in the non-PSD fraction is associated with a higher number of cytoskeletal and adhesion-related proteins (Fig. 4D). This is consistent with the ArfGAP family being involved in the remodeling of the actin cytoskeleton^35^. In particular, Agap2 has been implicated in the regulation of focal adhesion and neurite outgrowth^7^, suggesting that these functions correspond to Agap2 molecules localized in non-PSD fractions.

With respect to kinases, a large majority of those that are shared between the two different fractions include kinases involved in the CAMK family (Fig. 4F, Supplementary Fig. S8). There are also specific groups of kinases that reside in each particular fraction that can allow the inference of Agap2 function depending on its localization. For example, while the non-PSD fractions contain a number of kinases that can regulate cytoskeletal function (e.g. neurite outgrowth) such as Pkn2 and Dclk1^36, 37^, the PSD fraction has a larger number of PKA and PKC kinases. These families of kinases have been previously observed to associate mainly to upper and middle layers of PSD scaffolds, which are also associated to Agap2 in PSD complexes (Fig. 4F, Supplementary Fig. S8)^11^. Thus, while Agap2 has an overall capacity to interact with numerous proteins families, the spatial localization within neuronal compartments, restricts the interactions of Agap2 to a different set of protein-protein interactions and therefore its function within the PSD.

Because the main functional groups associated to GAPs and GEFs were those involved in GTP signaling, we also compared the clustering of Agap2 associated GAPs and GEFs in PSD and non-PSD complexes (Fig. 4G and H, Supplementary Fig. S8). Both Agap2 PSD and non-PSD complexes have their own respective RhoGAP and ArfGAP proteins. However, Agap2 PSD and non-PSD complexes shared the RasGAP, Dab2ip, which has been implicated in neurite outgrowth and cytoskeletal processes such as the Pkn2 and Dclk1 kinases in the non-PSD fraction^38^ (Fig. 4G, Supplementary Fig. S8). These processes can occur in PSD and non-PSD fractions and therefore the correspondent RasGAP effectors are localized in both fractions. Those that are not shared between the two cellular compartments include Syngap1, which is PSD-specific, along with Iqgap1, both of which are involved in the modulation of NMDA receptor signaling and synaptic plasticity^9, 39–41^ and therefore represent specific functions within PSD compartments. In contrast to PSD Agap2 complexes, which associate with a total of 7 GEF proteins, we were only able to identify a single GEFs in Agap2 non-PSD complexes (Fig. 4H, Supplementary Fig. S8). Thus, Agap2 present a differential clustering of GAPs and GEFs in PSD and non-PSD compartments, which might indicate a differential protein domain composition in their protein interactors.

Protein domain enrichment of Agap2 non-PSD interactors shows that they are also enriched in G_alpha and S_TKc domains (P = 4.76 × 10^−4^ and 1.75 × 10^−3^ for G_alpha and S_TKc, respectively, Bonferroni corrected), but also contained significant enrichment in SPEC domains (P = 7.30 × 10^−4^) which correspond to an increased quantity of cytoskeletal interactors outside of the PSD (Supplementary Table S4). Comparison of protein domain composition within Agap2 interactors located solely in the PSD again reflect the association of the PSD core-scaffolding machinery through the enrichment of SH3, PDZ, and PBPe domains (P = 5.62 × 10^−7^, 1.75 × 10^−5^, and 1.01 × 10^−3^ for SH3, PDZ, and PBPe, respectively). Surprisingly, a majority of the G-protein alpha subunit proteins found in Agap2 PSD complexes are found to be in common with Agap2 non-PSD complexes, leading to a significant enrichment of G_alpha domains among proteins in common (P = 1.21 × 10^−4^) (Supplementary Table S4).

### Interactions of Agap2, Syngap1, and Kalirin with risk factors of psychiatric disease

**Figure 5:**
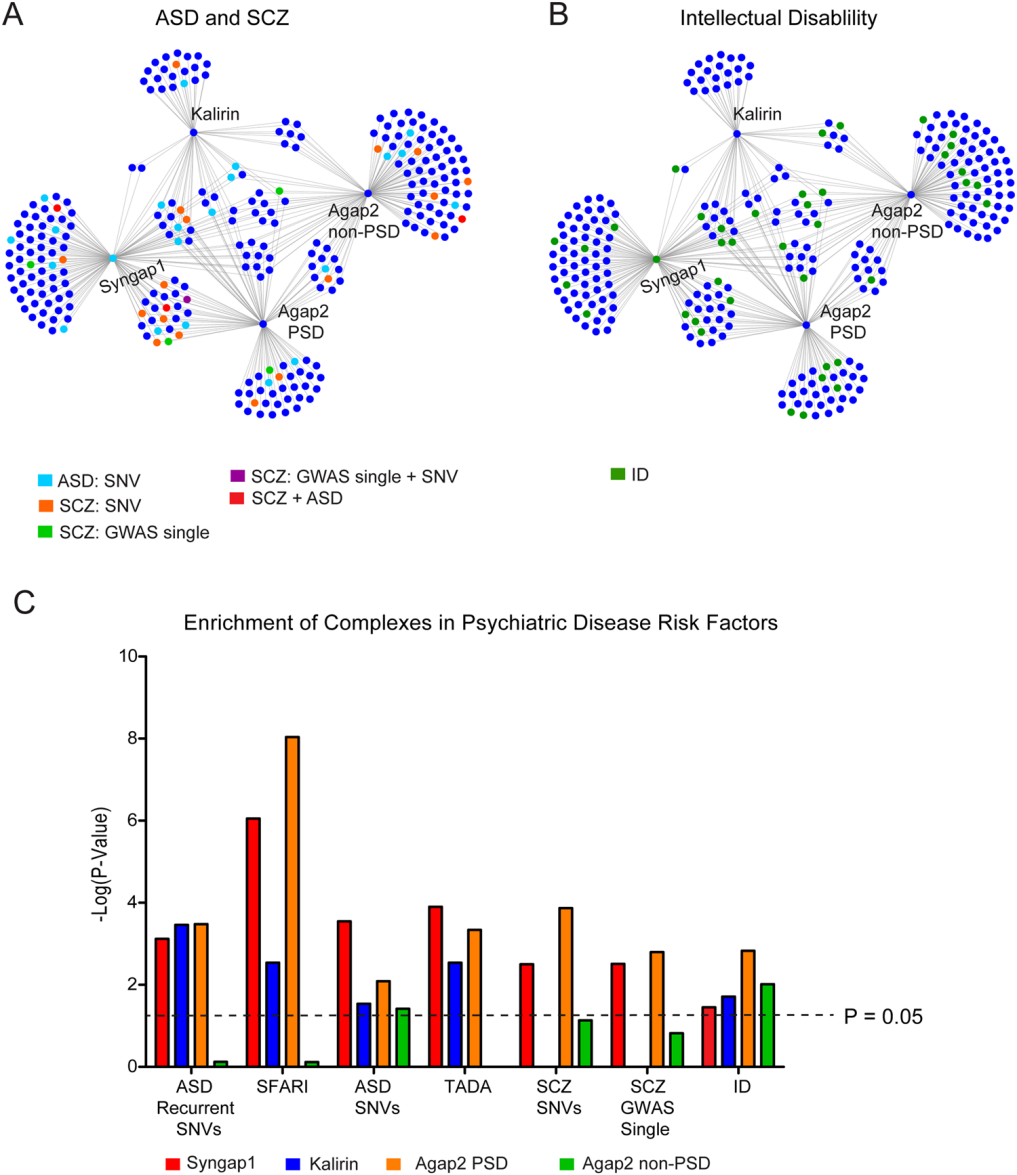
Distribution of Risk Factors for Psychiatric Disease within Protein-Protein Interaction Networks. (**A**) Distribution of risk factors implicated in contributing to autism spectrum disorder (ASD) and schizophrenia (SCZ) within the protein-protein interaction network of Syngap1, Kalirin, Agap2 within the PSD fraction, and Agap2 outside of the PSD. (**B**) Distribution of risk factors implicated in contributing to intellectual disability (ID) within the same protein-protein interaction network as in (**A**). (**C**) Enrichment of risk factors implicated in contributing to SCZ, ASD, and ID within Syngap1, Kalirin, Agap2 PSD, and Agap2 non-PSD complexes.

With Agap2, Syngap1, and Kalirin all being implicated in contributing to the risk of psychiatric disease^17–21, 42, 43^, we also mapped the distribution of SCZ and ASD risk factors (Fig. 5A, Supplementary Fig. S9) along with ID risk factors (Fig. 5B, Supplementary Fig. S9) within their protein-protein interaction networks. Over-representation analysis was performed in order to test for enrichment within different datasets for each psychiatric disease (Fig. 5C). Because of the heterogeneity of these disorders we tested risk factors implicated in Genome Wide Association Studies (GWAS), de novo mutations, single nucleotide variants, and recurrent variants.

We tested for the enrichment of genes implicated in ASD using the SFARI database^44^, single nucleotide variants^43^, recurring single nucleotide variants^17^, and a subset of 107 genes implicated in contributing to ASD through the TADA statistical model^45^ (Fig. 5C, Supplementary Table S5). All three PSD complexes were enriched in recurrent mutations (P < 0.05 for all, Bonferroni corrected) as these include several PSD-specific proteins such as Grin2b, Shank3, and Syngap1. Within the SFARI database, Syngap1 and Agap2 PSD complexes were highly enriched (P = 8.89 × 10^−7^ and 9.10 × 10^−9^ for Syngap1 and Agap2 respectively) followed by a decreased enrichment in Kalirin complexes (P = 0.02). In the single nucleotide variants gene list, Agap2 PSD and non-PSD complexes gained significance through the interaction of proteins with very little overlap between the two cellular compartments (P < 0.05 for all) (Fig. 5C). This shows that Agap2 interacts with different sets of risk factors contributing to ASD based on its subcellular localization and therefore its role in the pathophysiology of ASD cannot be circumscribed to only one synaptic compartment.

With respect to SCZ, we analyzed each protein complex for enrichment in single nucleotide variants and GWAS hits implicating a single loci. Contrary to ASD, SCZ risk factors showed a higher PSD component. In all cases, Syngap1 and Agap2 PSD complexes are highly enriched for SCZ risk factors (P < 4.0 × 10^−3^ for all). However, Kalirin and Agap2 non-PSD complexes, failed to reach significance (Fig. 5C). As the PSD complexes analyzed in this study interact with the core-scaffolding machinery, the results obtained for the ASD and SCZ gene lists is consistent with the hypothesis that the risk for these disorders is not spread throughout the entire PSD, but localized to this core machinery^11^. This is especially true for the Agap2 and Syngap1 complexes. However, the Kalirin complex which interacts with less members of the core-scaffolding complex, and therefore has a decreased association, is less significant in ASD and not significant in SCZ. In contrast to ASD and SCZ enrichment, all 4 complexes analyzed have similar levels of enrichment in known ID genes (P < 0.05 for all) (Fig. 5C). As seen for the ASD SNVs, Agap2 PSD and non-PSD complexes both have a very distinct set of interacting proteins that account for their significance of enrichment.

## Discussion

Through immunoisolation followed by HPLC-MS/MS we were able to determine protein complexes involving the interacting GAP/GEF proteins Agap2, Syngap1, and Kalirin which comprised a total of 281 interactions. In addition, we analyzed non-PSD Agap2 complexes (110 interactions), which was able to show functions of Agap2 based on differential localization. In analyzing the composition of GAP/GEF proteins complexes, we show for the first time that PSD GAPs and GEFs are clustered in subsets of protein complexes highly enriched in other GAPs and GEFs and that these protein complexes are associated to the PSD through its core-scaffold machinery.

While we and others have shown Syngap1 to interact with the PSD core-scaffolding machinery^11, 46, 47^ and Kalirin has been shown to interact with Dlg4^8^, here we are able to show that all three of the GAP/GEF proteins associate with multiple layers of the core-scaffolding machinery at the PSD. All of the complexes analyzed here also associate with glutamate receptors, reflecting their ability to relay signaling information from the receptors while relying on the scaffolding machinery for positioning.

In addition to the widespread interactions with the core-scaffolding machinery, the three PSD complexes were also enriched in the quantities of GAP and GEF proteins contained within each complex. This suggests that, GAP and GEF proteins are highly associated with and can cluster other GAPs and GEFs within protein interaction networks. In addition, Agap2 and Syngap1 complexes were also highly associated with G-protein subunit alpha proteins of the heterotrimeric G protein complexes with Agap2 complexes containing all four classes of G-protein alpha subunits and Syngap1 complexes mainly associated to the G-protein alpha subunit group I signature. This suggests a wide regulation of small G-protein signaling mechanisms by Agap2 and more specific modulation by Syngap1 at the PSD. In addition, all of the G protein alpha subunits within Agap2 PSD complexes were also shared with Agap2 non-PSD complexes. Thus, this represents a core functional unit within Agap2 complexes, while different interactors within each subcellular compartment provide the differential modulation of Agap2 function in a location-specific manner.

With Syngap1, Agap2, and Kalirin being individually implicated in psychiatric disease, here we show that their protein interactors are also highly enriched in recurrent mutations found in ASD. However, the enrichment for PSD complexes is not universal as Kalirin PSD complexes are not enriched and any SCZ gene set analyzed. While Agap2 non-PSD interactors are also not enriched in SCZ risk factors, Agap2 PSD and non-PSD complexes have similar levels of enrichment with respect to all ASD SNVs and genes implicated in contributing to ID. This reflects the diverse sets of Agap2 interactors implicated in contributing to psychiatric disease as the majority of interactors that contribute to the similar levels of enrichment between PSD and non-PSD complexes are unique to Agap2 based on its subcellular location. Furthermore, protein interactions contributing to disease relevant aspects of Agap2 have been verified within this study. While we have shown that the interactomes of baits implicated in contributing to psychiatric disease are enriched with other psychiatric disease risk factors, this concept has also been shown to be true with respect to neurodegenerative diseases^48^. For example, the interactomes of the Alzheimer’s Disease (AD) risk factors, APP and PSEN1, have been shown to be significantly associated to AD risk factors identified through GWAS data^48^. This provides evidence for the concept that proteins linked to a particular disease may be closer together within protein-protein interaction networks than by chance^49^.

Recently, increased Agap2 expression have been associated to the impaired signaling, synaptic function, and behavior in observed in fragile X syndrome (FXS)^33^. It was proposed that Agap2 contributed in the dysregulation of metabotropic glutamate receptors 1/5 (Grm1/5) signaling observed in FXS. Our results show that Agap2 can associate not only to Grm1 and 5, but also to type II Grm2 and 3, suggesting a wide regulation of metabotropic glutamate signaling by Agap2. Moreover, Agap2 was also able to interact with Fmr1 binding partners Cyfip1 and Cyfip2, indicating that Agap2 might modulate Fmr1 function not only at the level of glutamate receptor inputs but also through protein-protein interactions within the Fmr1 protein interaction network. A number of these protein interactions were also identified in non-PSD fractions, including Grm1, Grm2 and Cyfip2. In addition, we were also able to identify Fmr1 as an Agap2 protein interactor in non-PSD fractions, confirming a role of Agap2 in the Fmr1 protein network. Interestingly, recent studies have also suggested that altered signaling pathways observed in Syngap1 mutation mice, might converge with FXS^50^. In line with this, we also identified Grm5 and Cyfip2 as Syngap1 interactors, suggesting a convergence in a protein interaction network within glutamate metabotropic receptors, synaptic Agap2, and Fmr1 protein interactors.

We have identified numerous interactors of PSD GAPs and GEFs and provide an example of how interacting proteins can change based on cellular compartmentalization. This shows that GAPs and GEFs may play roles in multiple cellular processes and future studies will need to address the role of GAPs and GEFs not in isolation but within protein interaction networks. This will allow the investigation of how disease-relevant mutations affect not only their activity, but also the composition of protein interactions within the synapse.

## Materials and Methods

All experimental protocols used in this study were approved by the University of Southern California (USC) Institutional Animal Care and Use Committee (IACUC), protocol number 11782-CR003. All methods were carried out in accordance with relevant guidelines and regulations.

### Postsynaptic Density Preparations

Postsynaptic density preparations were performed as described^33, 51^. Briefly, adult mouse (3–4 months in age) cortex was homogenized in sucrose buffer (0.32 M sucrose, 10 mM Hepes buffer (pH 7.4), 2 mM EDTA, 30 mM NaF, 20 mM β-glycerol phosphate, 5 mM sodium orthovanadate, and Roche cOmplete protease inhibitor cocktail) and centrifuged at 500 *g* for 6 minutes. Supernatant was collected and then spun at 10,000 g for 10 minutes. The resulting pellet was solubilized in triton buffer (50 mM Hepes (pH 7.4), 2 mM EGTA, 2 mM EDTA, 50 mM NaF, 20 mM β-glycerol phosphate, 5 mM sodium orthovanadate, Roche cOmplete, and 1% Triton X-100. The solubilized pellet was centrifuged at 30,000 rpm for 30 min and supernatant was collected for non-PSD fractions. The resulting pellet was collected and solubilized in DOC buffer (50 mM Tris (pH 9), 30 mM NaF, 5 mM sodium orthovanadate, 20 mM β-glycerol phosphate, 20 µM ZnCl_2_, Roche complete, and 1% sodium deoxycholate) and served as the PSD fraction.

### Immunoprecipitation

Immunoprecipitation experiments were performed as previously described^11, 49^. Lysate containing 2 mg of total protein was incubated with the indicated primary antibody at a concentration of 1 µg/µl at 4 degrees Celsius overnight with rotation. The following day, IPs were incubated with Dynabeads protein G (Novex) for 2 hours at 4 degrees Celsius with rotation. IPs were washed three times with IP wash buffer (25 mM Tris (pH 7.4), 150 mM NaCl, 1 mM EDTA, and 1% Triton X-100 or sodium deoxycholate where appropriate). IPs were re-suspended in 2X LDS sample buffer and incubated at 95 degrees Celsius for 15 minutes to elute protein complexes. The elutant was incubated with DTT at a final concentration of 1 mM at 56 degrees Celsius for 1 hour followed by incubation with Iodoacetamide at a final concentration of 20 mM at room temperature for 45 minutes. Primary antibodies used for immunoprecipitation in this study included PIKE (Agap2) (Bethyl Laboratories, catalog #A304-262A), Kalirin (Millipore, catalog #07-0122), Syngap1 (Cell Signaling Technologies, catalog #5539), Rabbit IgG Isotype Control (ThermoFisher, catalog #06-6102) and GST (NeuroMab, catalog #75-148). All antibodies were used as obtained from the manufacturer.

### Mass spectrometry sample preparation

Samples were prepared for mass spectrometry as described in Li *et al*.^11^. Briefly, samples were separated on 4–12% Bis-Tris gels (NuPAGE Novex) followed by staining with InstantBlue (Expedeon) for protein visualization. Following destaining, lanes were cut and placed in 96-well perforated plates for destaining and peptide digestion via trypsin at 37 degrees Celsius overnight. Peptides were then extracted with acetonitrile, dried down using a Savant SPD 1010 SpeedVac Concentrator (Thermo Scientific), and then suspended in 3% ACN/0.1% FA. A Nano/Capillary LC System Ultimate 3000 (Thermo/Dionex) was used for desalting and reverse-phase separation of peptides. The LC system was coupled to a hybrid linear ion-Fourier transform ion cyclotron resonance LTQ-FT (FTICR) 7 Tesla mass spectrometer (LC/MS) for data acquisition.

### Data analysis

Proteome Discoverer 1.4 (Thermo Scientific) was used to process MS data which was analyzed using both the Sequest and Mascot V2.4 (Matrix science) against a modified mouse database from Uniprot combined with its decoy database. With respect to analysis settings, the mass tolerance was set 10 parts per million for precursor ions and 0.8 daltons for fragment ions, no more than two missed cleavage sites were allowed, static modification was set as cysteine carboxyamidation, and dynamic modification was set as methionine oxidation. False discovery rates (FDRs) were automatically calculated by the perculator node of Proteome Discoverer and a peptide FDR of 0.01 was used for cut-offs. Peptides with high confidence were considered as true hits and proteins with at least two different peptides were accepted. Protein Interactions were considered positive if a minimum of two peptides were present in at least two assays and absent in anti-GST controls. The datasets generated and/or analyzed during the current study have been deposited to the ProteomeXchange Consortium via the Proteomics IDEntifications (PRIDE) partner repository under the dataset identifier PXD006326^52^.

### Western blotting

Immunoprecipitation was carried out as described above in non-PSD (triton soluble) for Agap2 and PSD (DOC soluble) fractions for Agap2, Kalirin. and Syngap1. For conformation of protein interactions, total lysate (20 ug) and a control rabbit IgG immunoprecipitation were ran alongside Agap2, Kalirin, or Syngap1 immunoprecipitation experiments. Protein was loaded onto 4–12% Bis-Tris gels (NuPAGE Novex) and separated at 135 V for 1.5 hours. Proteins were transferred to a PVDF membrane using a BIO-RAD Trans Blot Turbo System. Membranes were then blocked using 5% bovine serum albumin (BSA) in 0.05% TBST (TBS-Tween 20) for 1 hour at room temperature and then incubated with the primary antibody at a 1:1000 dilution overnight at four degrees Celsius. Primary antibodies include PIKE (Agap2) (Bethyl Laboratories, catalog #A304-262A), Syngap1 (Cell Signaling Technologies, catalog #5539), Kalirin (Millipore, catalog #07-0122), Camk2a (ThermoFisher, catalog #13-7300), Cit (Bethyl Laboratories, catalog #A302-303A), Dlg4 (ThermoFisher, catalog #MA1-045), Fmr1 (abcam, catalog #ab17722), Git1 (NeuroMab, catalog #75-094), NR2B (NeuorMab, Catalog #75-097), Gsk3β (Cell Signaling Technologies, catalog #9832), mGluR2 (Cell Signaling Technologies, catalog #76012), mGluR5 (Cell Signaling Technologies, catalog #55920), Nckap1 (Proteintech, catalog #12140-1-AP), and Tnik (Bethyl Laboratories, catalog #A302-695A). All antibodies were used as obtained from the manufacturer. The following day, membranes were washed with 0.05% TBST four times for 10 minutes each, incubated with the respective secondary antibody for 1 hour, and subsequently washed 4 time for 5 minutes each. Western blots were incubated with Pierce ECL Western Blotting Substrate (Thermo Scientific) for 5 minutes and then imaged using a Carestream Image Station 4000 MM Pro.

### Over-representation analyses

Enrichment of GAP and GEF domain containing proteins within PSD protein complexes was carried out using the one-tailed Fisher’s exact test with the option “greater,” followed by the Bonferroni correction for multiple comparisons using the (fisher.test) and (p.adjust) packages in R, respectively. The total number of proteins within the PSD served as the background.

SMART protein domain enrichment was completed using the Database for Annotation, Visualization, and Integrated Discovery (DAVID) version 6.8^53^ using the default settings and the number of protein coding genes expressed in purified mouse cortical neurons derived from Zhang *et al*.^54^ as the background for each gene list analyzed. To obtain the background, we accessed processed transcriptomic data from NCBI Gene Expression Omnibus (GEO) accession number GSE52564 and compiled a list of genes that were consistently expressed at a statistically significant level as defined by Zhang *et al*.^54^ (FPKM > 0.1) between biological replicates. After filtering out non-coding genes, this resulted in a list of 13,761 protein coding genes. Only domains that reached statistical significance following Bonferroni correction of multiple comparisons are reported in the text.

For disease gene set enrichment, we incorporated several different lists encompassing genes that are likely to contribute to autism spectrum disorder (ASD), schizophrenia (SCZ), and intellectual disability (ID). These included: (1) Supplementary Table 15 from Turner *et al*.^17^ which encompasses a list of 57 genes found to have recurrent de novo likely gene disrupting (LGD) mutations in probands with autism from a collection of exome sequencing studies^43, 45^, (2) Supplementary Table 7 from Iossifov *et al*.^43^ which contains 353 genes harboring validated de novo LGD mutations in probands with autism, (3) Supplementary Table 3 from De Rubeis *et al*.^45^ which included 107 genes with an FDR < 0.3 implicated in contributing to autism through the TADA statistical model, 4) SFARI gene database^44^ as of February 2016 which was restricted genes containing the SFARI gene rankings 1 – 4 (strong evidence to minimal evidence) and S (syndromic) (5) Supplementary Table 5 from Fromer *et al*.^14^ which contains de novo SNVs identified in probands with SCZ excluding silent mutations, (6) Supplementary Table 3 from Ripke *et al*.^55^ restricted to loci reaching genome wide significance in patients with SCZ and implicating a single gene, (7) Supplementary Table 4 from Leliveld *et al*.^56^ which contains a list of 1537 genes known to contribute to ID. For all tests, protein coding genes expressed in purified mouse cortical neurons derived from Zhang *et al*.^54^ served as the background measured by one-tailed Fisher’s exact test with the option “greater,” followed by the Bonferroni correction for multiple comparisons using the (fisher.test) and (p.adjust) packages in R, respectively.

## Electronic supplementary material

Supplementary Information

Supplementary Table S1

Supplementary Table S2

Supplementary Table S3

Supplementary Table S4
