# Supplementary material for: Synaptic GAP and GEF Complexes Cluster Proteins Essential for GTP Signaling
Source: Sci Rep. 2017 Jul 13;7:5272. doi: 10.1038/s41598-017-05588-3 (PMC5509740; doi:10.1038/s41598-017-05588-3)
Supplement: Supplementary file 1 — Supplementary Information [file 41598_2017_5588_MOESM1_ESM.pdf]

Supplementary Information

Synaptic GAP and GEF Complexes Cluster Proteins Essential for GTP Signaling

Brent Wilkinson[1], Jing Li[1], Marcelo Coba[1, 2, *]

[1] Zilkha Neurogenetic Institute, Keck School of Medicine, University of Southern California, Los Angeles, CA 90033, USA

[2] Department of Psychiatry and Behavioral Sciences, Keck School of Medicine, University of Southern California, Los Angeles, CA 90033, USA

[*] Correspondence should be addresses to: coba@usc.edu

Figure S1

Figure 2a

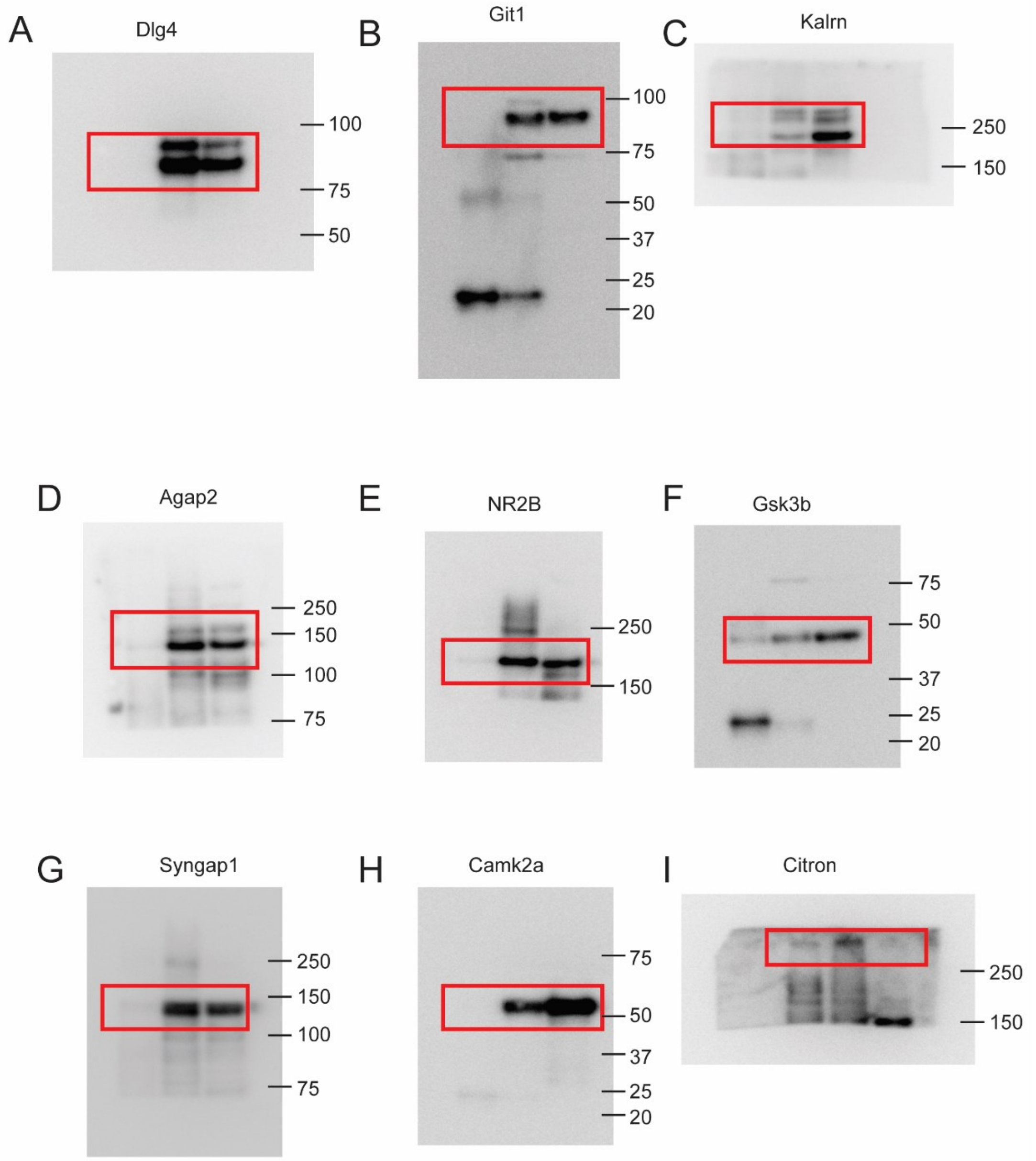

Supplementary Figure 1. Full length images of western blots from figure 2A. Indicated molecular weight markers are in kilodaltons (KDa). Cropped portions are indicated by red boxes.

Figure S2

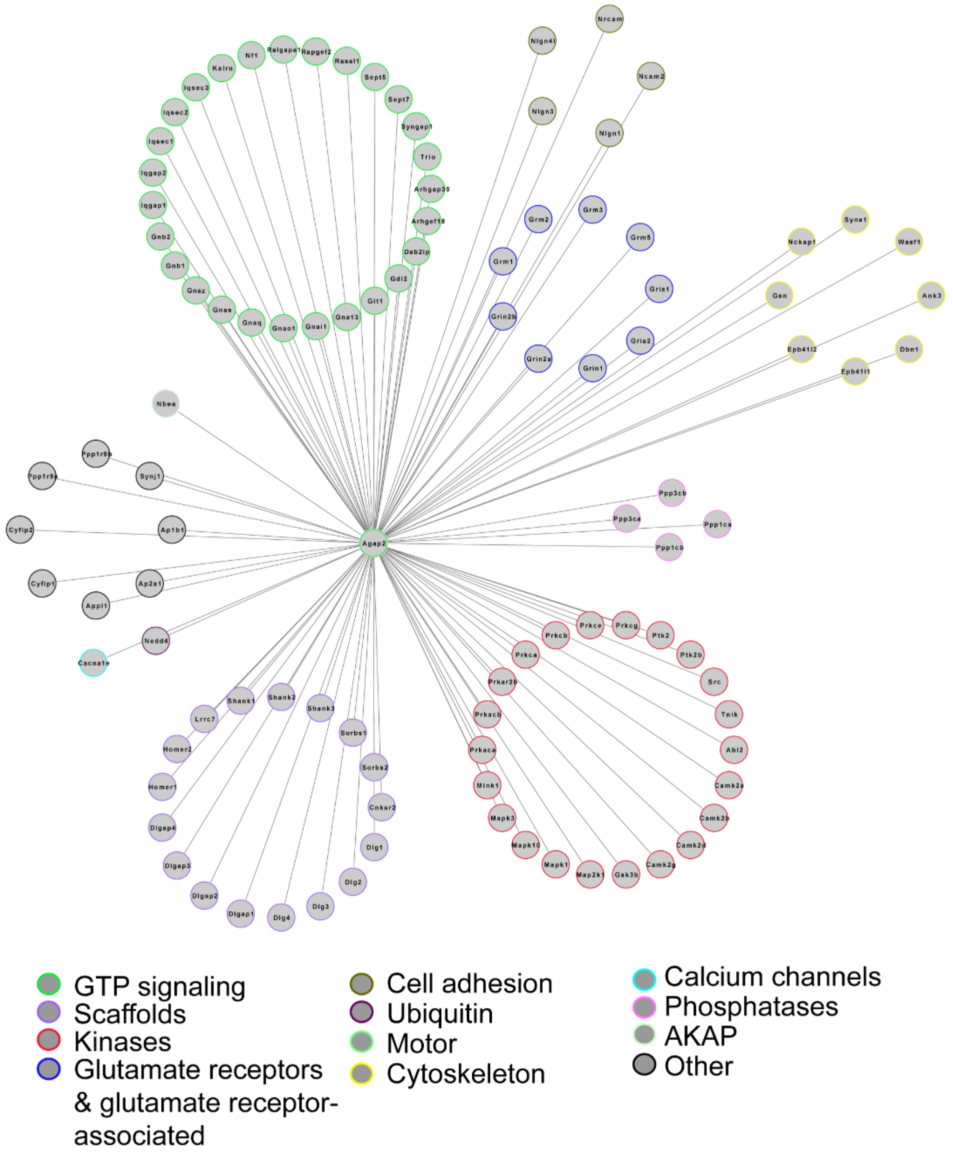

Supplementary Figure 2. Expanded interactome of Agap2-PSD shown in figure 2B clustered by protein function.

Figure S3

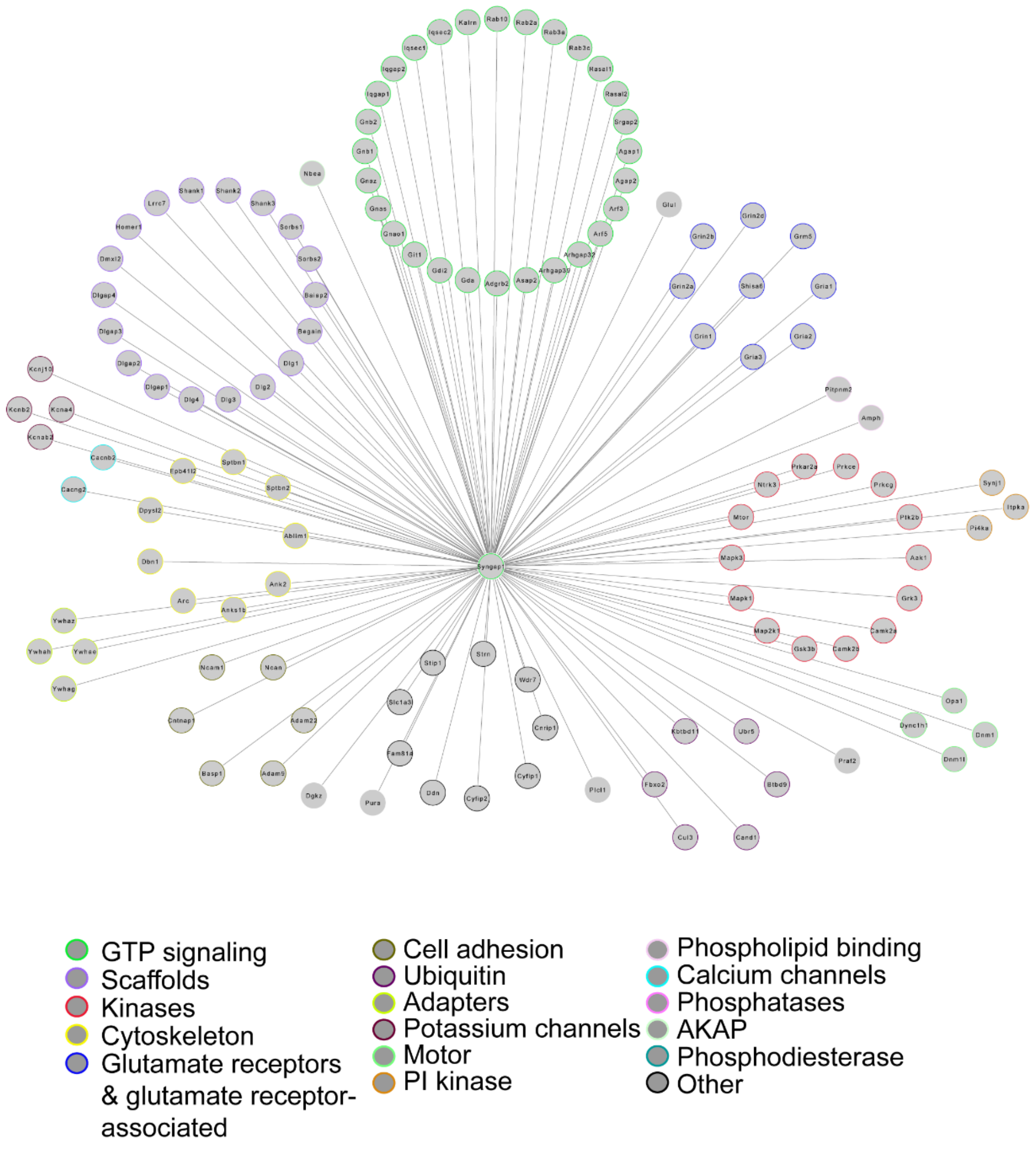

Supplementary Figure 3. Expanded interactome of Syngap1-PSD shown in figure 2D clustered by protein function.

Figure S4

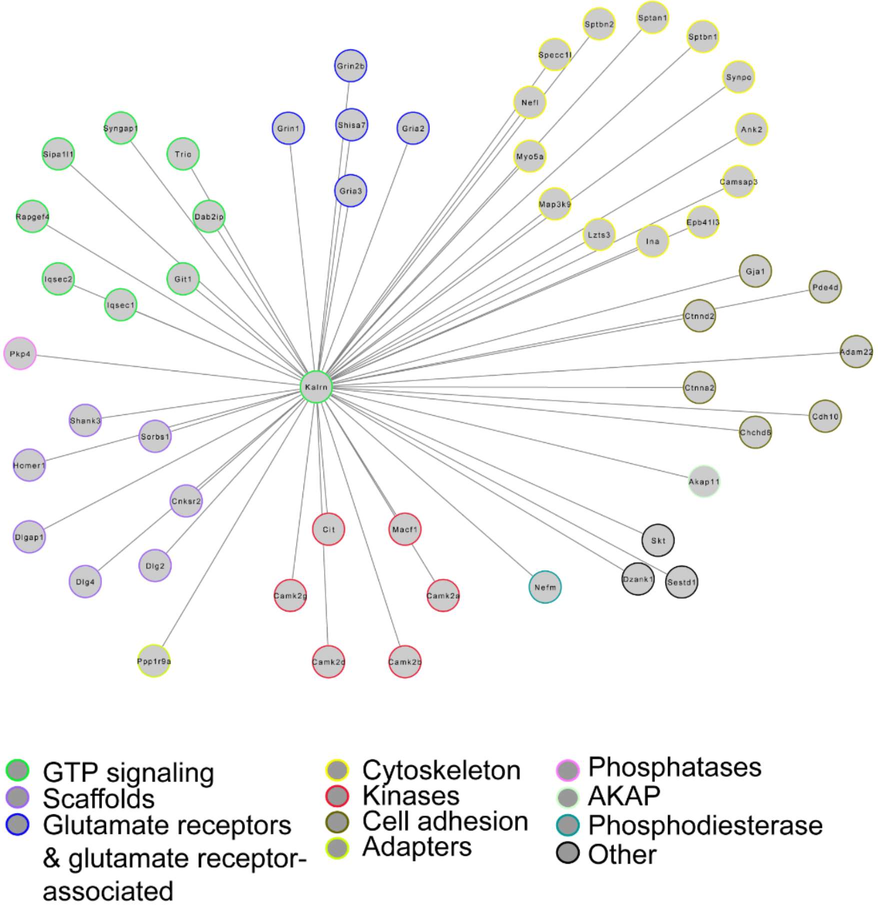

Supplementary Figure 4. Expanded interactome of Kalirin-PSD shown in figure 2F clustered by protein function.

Figure S5

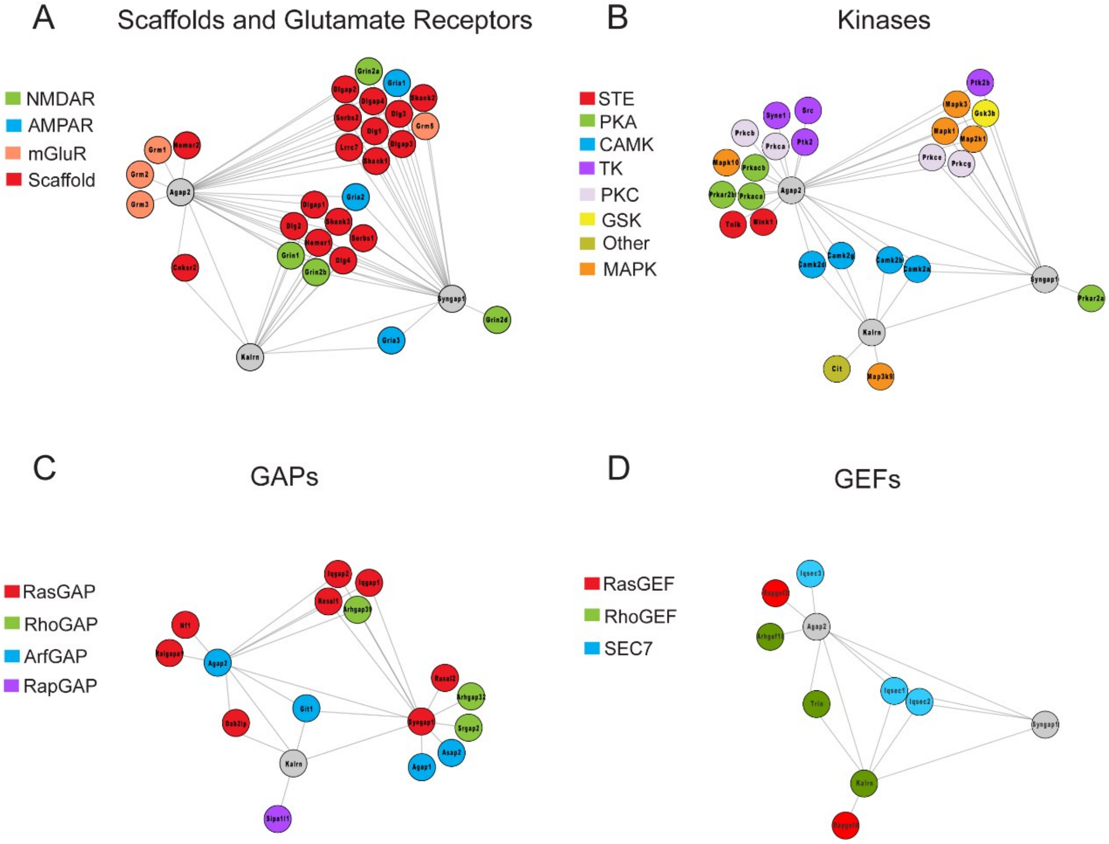

Supplementary Figure 5. Expanded sub-networks of highlighted nodes in PSD interactomes shown in figures 3A – D.

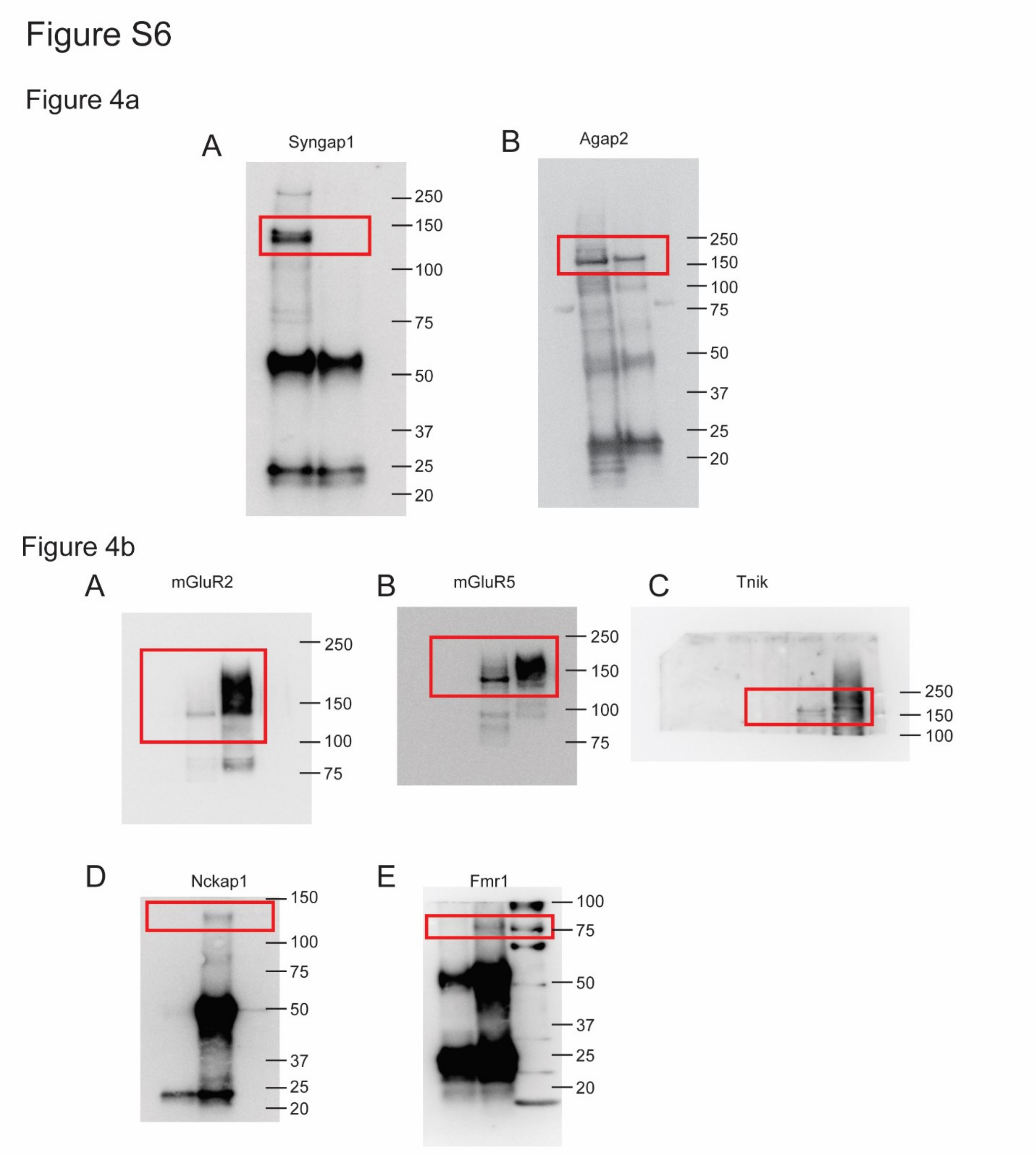

Supplementary Figure 6. Full length images of western blots from figure 4A and B. Indicated molecular weight markers are in kilodaltons (KDa). Cropped portions are indicated by red boxes.

Figure S7

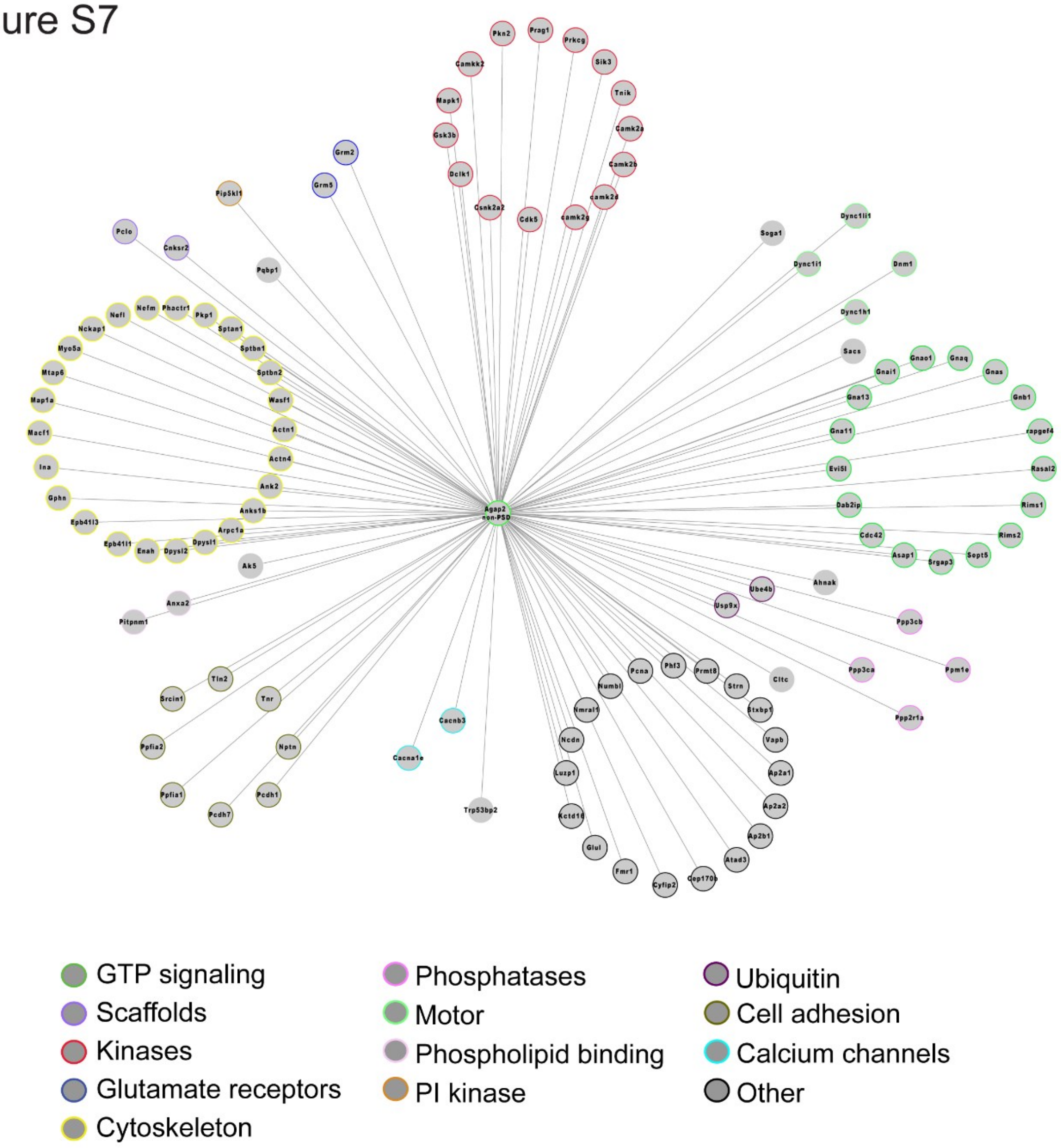

Supplementary Figure 7. Expanded interactome of Agap2 non-PSD shown in figure 4C clustered by protein function.

Figure S8

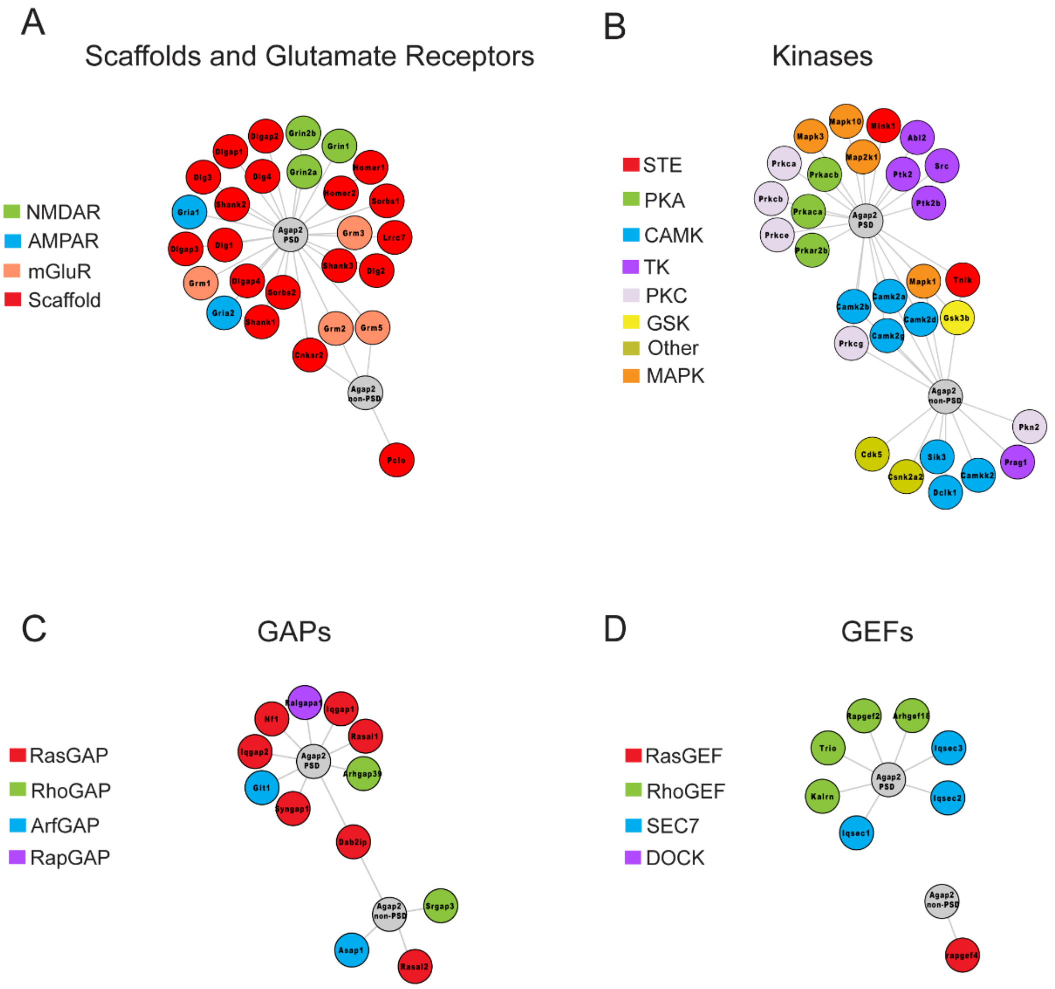

Supplementary Figure 8. Expanded sub-networks of highlighted nodes in PSD versus non-PSD interactomes shown in figures 4E – H.

Figure S9

A

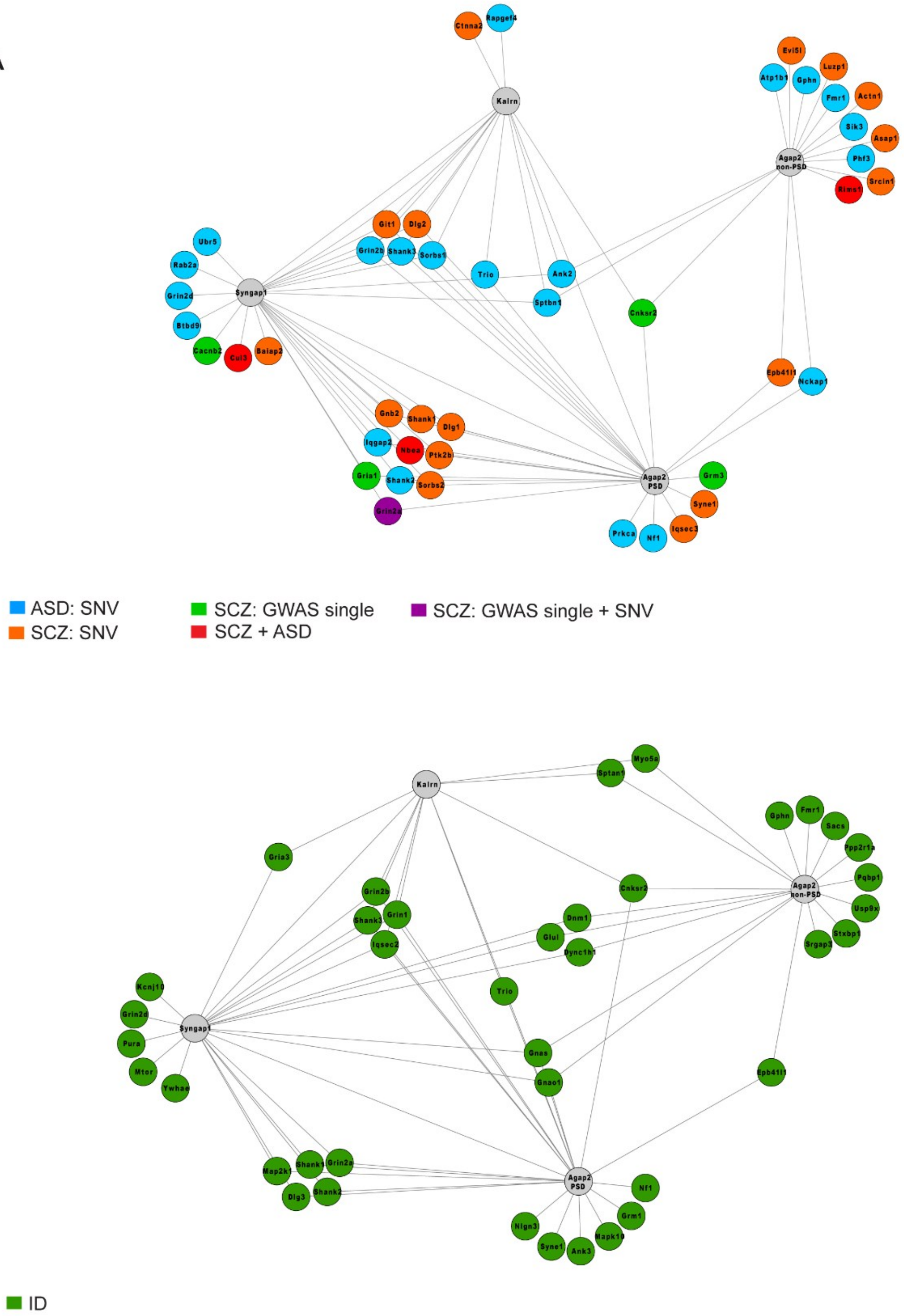

Supplementary Figure 9. Expanded sub-networks of highlighted nodes representing psychiatric disease risk factors in interactomes shown in figures 5A and B.

Legends for external supplemental tables:

Supplementary Table S1. PSD proteins from mouse cortex identified via HPLC-MS/MS. Table contains proteins identified in PSD fractions via HPLC-MS/MS in mouse cortex. Identified protein contain their associated gene symbol, associated mouse genome informatics (MGI) database ID, and associated gene name.

Supplementary Table S2. Domain Architecture of GAPs and GEFs at the PSD. Table contains GAP and GEF proteins identified in PSD fractions of adult mouse cortex along with their associated protein domain structure. Each protein contains their associated gene symbol, MGI ID, associated gene name, the GAP or GEF domain present within the respective protein, followed by other protein domains co-occurring with the GAP or GEF domains.

Supplementary Table S3. GAP/GEF Protein Interactions and Functional Annotation. Table contains interactions Agap2, Syngap1, and Kalirin within the PSD fractions along with Agap2 in non-PSD fractions determined via HPLC-MS/MS. Interactors of each target protein followed by tables exported from Proteome Discoverer are listed on separate tabs. For each target protein, the interacting proteins contain their associated gene symbol, MGI ID, and functional annotation. Functional annotation of interactors corresponds to figures 2 and 4.

Supplementary Table S4. SMART domain enrichment within PSD complexes. Table contains results of enrichment analyses of protein domains within protein complexes determined in this study according to the SMART protein domain database. Separate analyses are contained on individual tabs named according to the target protein. Results were obtained using the DAVID database.

Supplementary Table 5 - Statistical Enrichment of Genes Implicated in Contributing to Psychiatric Disease				
**Dataset**	**Syngap1**	**Kalirin**	**Agap2 PSD**	**Agap2 non-PSD**
Turner et al., 2016 - Supplementary Table 16	7.63E-04	3.44E-04	3.31E-04	4.66E-01
SFARI Database	8.89E-07	2.86E-03	9.10E-09	4.64E-01
Iossifov et al., 2014 - Supplementary Table 7	2.80E-04	2.88E-02	8.17E-03	4.70E-02
De Rubeis et al., 2014 - Supplementary Table 3 - TADA	1.27E-04	2.91E-03	4.59E-04	1.00E+00
Fromer et al., 2014 - Supplementary Table 5	3.14E-03	1.00E+00	1.34E-04	1.01E-01
Ripke et al., 2014 - GWAS signale indicating a single gene	3.09E-03	1.00E+00	1.58E-03	1.87E-01
Lelieveld et al., 2016 - Supplementary Table 4	3.57E-02	1.16E-02	1.47E-03	9.69E-03

Supplementary Table S5. Statistical Enrichment of Genes Implicated in Contributing to Psychiatric Disorders. Table contains results of over-representation analyses of lists of genes implicated in contributing to autism spectrum disorder, schizophrenia, and intellectual disability within the protein complexes determined in this study. Results were obtained using the one-tailed Fisher's exact test followed by the Bonferroni correction for multiple comparisons.
